# Microbiome Related Cytotoxically Active CD8+ TIL Are Inversely Associated With Lung Cancer Development

**DOI:** 10.3389/fonc.2020.531131

**Published:** 2020-12-09

**Authors:** Leliang Zheng, Jiaqi Xu, Buqing Sai, Yinghong Zhu, Lujuan Wang, Na Yin, Fenglei Yu, Wen Zhou, Minghua Wu, Jingqun Tang, Juanjuan Xiang

**Affiliations:** ^1^ Hunan Cancer Hospital, The Affiliated Cancer Hospital of Xiangya School of Medicine, Central South University, Changsha, China; ^2^ Department of Thoracic Surgery, The Second Xiangya Hospital, Central South University, Changsha, China; ^3^ Cancer Research Institute, School of Basic Medical Science, Central South University, Changsha, China; ^4^ NHC Key Laboratory of Carcinogenesis and the Key Laboratory of Carcinogenesis and Cancer Invasion of the Chinese Ministry of Education, Xiangya Hospital, Central South University, Changsha, China; ^5^ Hunan Key Laboratory of Nonresolving Inflammation and Cancer, Changsha, China; ^6^ Hunan Key Laboratory of Early Diagnosis and Precise Treatment of Lung Cancer, Changsha, China

**Keywords:** *Pasteurella*, therapeutic vaccine, microbiome, lung cancer, TIL

## Abstract

Lung cancer is the most common cancer type around the world. Although major advances in cancer therapy, lung cancer has been the largest proportion of all cancer-related deaths. The respiratory tract contains many types of bacteria and a distinct lung microbiome in lung cancer patients was described in many studies. The specific roles of these lung microorganisms in lung cancer progression remain unclear. In this study, we evaluated the effect of inhalation of bronchoalveolar fluid (BAL) in the lung cancer cell growth. The microbiome-based immune and carcinogenesis was examined in tumor-bearing mouse model. We found that inhalation of BAL collected from non-small cell lung cancer (NSCLC) patients altered the lung microbiota and inhibited tumor cell growth. The inhibitory effect was due to the infiltration of CD3 and CD8^+^ T cells and decrease of M2 macrophages in lungs. The microbial communities of NSCLC BAL inhalation group were dominated by *Pasteurella*, whereas the microbial communities of non-cancer control and PBS inhalation group were dominated by *Delftia*. Linear discriminant analysis (LDA) indicated that the genera *Pasteurella*, *Pseudomonas*, and *Chryseobacterium* were increased in NSCLC BAL inhalation group, while genera *Delftia*, *Ezakiella*, *Blautia*, *Cloacibacterium*, and *Microvirga* et al. were increased in PBS and Non-cancer group. We demonstrated a significant positive correlation between *Pasteurella* and cytotoxic CD8^+^ TIL and a negative correlation with M2 macrophages. *Coriobacteriaceae* was positively correlated with M2 macrophages and negatively correlated with CD8^+^ cells. The abundance of *Pasteurella* was negatively correlated with tumor cell growth. Our findings provide a promising strategy that can be used as a therapeutic vaccine for lung cancer patients.

## Introduction

Lung cancer is the leading cause of cancer-related deaths (1). Although therapeutic strategies for lung cancer including surgical resection, chemotherapy and targeted therapies have made significant advances, it remains one of the deadliest cancers in humans (2, 3). Numerous vaccine strategies that represent an option for cancer immunotherapy are currently being evaluated (4). A viral-based vaccine TG4010 plus chemotherapy improve progression-free survival in non-small cell lung cancer (NSCLC) patients (5).

Lungs were previously considered to be sterile (6). Culture-independent methods based on nucleic acid 16S rRNA sequences show that the lower respiratory tract contained many types of bacteria. A distinct lung microbiome in lung cancer patients was described in many studies (7–9). Potential mechanisms by which microbiota affect lung cancer can be divided into three categories: creation of an inflammatory milieu, metabolic effect of dysbiosis, and genotoxicity (8). Dysbiosis occurs when harmful microbes take over the environment. *Capnocytophaga* and *Veillonella* that reside in oral were significantly higher in lung cancer patients (10). *Veillonella* and *Megasphaera* were relatively more abundant in bronchoalveolar fluid (BAL) from lung cancer patients, which represents the lower respiratory tract environment (11). However, the specific roles of these microorganisms that reside in respiratory tract in lung cancer progression remain unclear.

The relationship between cancer and inflammation has long been discussed. Inflammation can be classified as acute inflammation, which is a positive response, and chronic inflammation, which has been implicated in cancer progression (12). Infectious or non-infectious tissue damage associated with chronic inflammation predispose to cancer (13, 14). Chronic viral, bacterial, and parasitic infections have been implicated in a variety of cancer type (15). The inflammatory microenvironment, angiogenesis, proinflammatory cytokines contribute to the cancer progression. On the contrary, an inverse association between acute infections and cancer development has long been noticed since 1891, when William Coley used extracts from bacteria to induce tumor regression (15).

In this study, our findings showed that distinct composition of microbiota was related to the cancer progression. High abundance of *Pasteurella* in the lung biopsies led to significant inhibition of tumor growth in tumor-bearing mice. We demonstrated that the inhibitory effects can be dictated by CD8+ T cell infiltration. Our research provides a clue for bacteria-based vaccine treatment for lung cancer.

## Materials and Methods

### Patient and Sample Characteristics

The BAL was collected from eight individuals without smoking history, including with four NSCLC patients with clinical stage 1 and four non-cancer controls. BAL was collected by instillation and aspiration of 20 ml of 0.9% NaCl from bronchoscope. These patients have not been treated with antibiotics. Patients with clinical evidence of infection, sepsis, or active tuberculosis were excluded. The patients were informed of the sample collection and signed informed consent forms. The collection and use of samples were approved by the ethical review committees of the second Xiangya Hospital, Central South University.

### Animal Experiments

Thirty-four six-week-old male C57BL/6 mice were used to examine allograft tumor growth. All animal experiments were conducted following protocols approved by Central South University, China. Mice were intraperitoneally injected with cefoperazone-sulbactam at 30 mg/kg for 5 days. The BAL collected from each individual was given three times to mice by aerosol inhalation. Three to four mice each group was treated with 5–6 ml of BAL each time simultaneously in aerosol inhalation device. Seven days after aerosol inhalation, the mice were intravenously injected with luciferase expressing syngeneic murine Lewis Lung Cancer cells (luci-LLC cells). The cancer cell nodules formed in the lung were measured by H&E staining on lung paraffin sections, or detected by *ex vivo* luciferase based non-invasive bioluminescence imaging using IVIS Lumina II (PerkinElmer).

### Flow Cytometry

Lungs were minced, digested, and homogenized. Cells were passed through a 70-um nylon filter. After the washing with PBS, red blood cells from lungs were lysed by 5 min incubation in RBC lysing buffer (555899, BD, USA). The cells were incubated with fluorochrome-conjugated antibodies that recognize extracellular epitopes included CD4 (550954, BD, USA), CD8 (553032, BD, USA), CD11B (550993, BD, USA), and CD11C (117308, BD, USA). M1 and M2 macrophages were identified as CD11C^+^/CD11B^+^ or CD11C^+^/CD206^+^cells respectively. For M2 macrophage staining, the cells were treated with eBioscience^TM^ Permeabilization Buffer (00-5523-00, ThermoFisher Scientific, USA) and stained for intracellular antigens CD206 (141707, BD, USA). Cells were then washed and analyzed on a BD FACS Caliber.

### Immunofluorescence

The lung tissue was prepared into paraffin sections. Fixed samples were hybridized and antigen repair, followed by incubating with fluorochrome-conjugated antibodies that recognize extracellular epitopes included CD3(100204, Biolegend, USA) and CD8(553032, BD, USA). After staining, slides were mounted with Fluormount G, and examined on a Fluorescence microscope. Images were analyzed with ImageJ software.

### Lung Microbiota Analysis

Lung biopsies were collected after execution of mice. Genomic DNA was extracted from the lung biopsies and 16S rRNA gene was amplified by PCR. The sequencing of the PCR products was performed on an illumine Miseq platform (Allwegene co. China). Sequences were aligned and compared to NCBI BLAST. The species accumulation curve was used to estimate the number of species in the lungs of mice. The difference in the lung microbiota was analyzed by Principal Component Analysis of OTUs and analysis of similarities (ANOSIM). The differentially abundant genera between groups was identified by LEfSe analysis. The raw data for this study (16s rRNA sequencing) can be found in the (SRA, PRJNA672792, NCBI).

### Statistical Analysis

The statistical analyses except as noted were performed using GraphPad Prism 6.0 (GraphPad Software, San Diego, CA, USA). Data of flow cytometric analysis are presented as the mean ± S.D. from at least three separate experiments. Group comparisons were performed using student t test. A p value of less than 0.05 was considered to be significant.

## Results

### BAL From Lung Cancer Patients Inhibited Growth of Lung Cancer Cells

**Figure 1:**
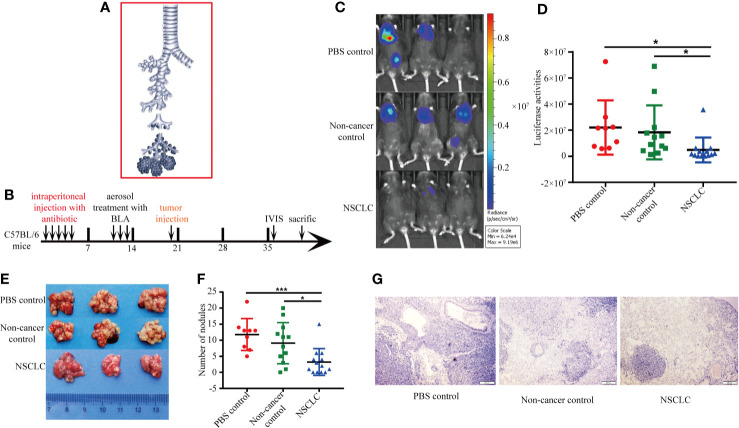
BAL from lung cancer patients inhibited growth of lung cancer cells. **(A)** BAL was collected from bronchial and alveolar spaces. **(B)** Experimental protocol for construction of animal model and treatment scheme. The C57BL/6 mice were pretreated with broad-spectrum antibiotics cefoperazone before aerosolizing BAL. Five days after aerosolization, synergistic luci-LLC cells were intravenously injected into the mice. The *in vivo* imaging was used to measure the growth of cancer cells in lungs. **(C)** Bioluminescent images were shown. **(D)** Activities of luciferase were shown in scatter diagram. **(E)** Lung metastatic nodules from mice. **(F)** The number of metastatic tumor modules formed in the lungs was counted and displayed in scatter plot. Data were analyzed with Student’s t test, *p < 0.05, ***p < 0.001. **(G)** HE staining of lung section.

In order to investigate the roles of BAL in the cancer progression, we aerosolized BAL from eight people including non-cancerous control and lung cancer patients to the C57BL/6 mice. BAL was collected from bronchial and alveolar spaces (**Figure 1A**). The treatment protocol was shown in **Figure 1B**. The C57BL/6 mice were intraperitoneally injected with broad-spectrum antibiotics cefoperazone for one time per day for 5 days before aerosolizing BAL. An aerosol from BAL was generated by an aerosolizing inhalation device with a nozzle. The mice were divided into three groups: non-cancer control group was aerosolized with BAL from non-cancer controls one time per day for 3 days (Group A: n = 12; three mice for each patient); NSCLC group was aerosolized with BAL from NSCLC patients one time per day for 3 days (Group B, n = 13; three to four mice for each patient); PBS group was aerosolized with PBS one time per day for 3 days (Group C: n = 9). Seven days after aerosolization, synergistic luci-LLC cells were intravenously injected into the mice (**Figure 1B**). The *in vivo* imaging was used to measure the growth of cancer cells in lungs. We found that aerosolizing BAL from NSCLC patients inhibited the growth of lung cancer cells compared to aerosolizing BAL from non-cancer controls or PBS controls (**Figures 1C, D**). The number of tumor modules formed in the lungs of mice was greater in PBS group and non-cancer control group (**Figures 1E, F**). Hematoxylin and eosin-stained sections showed tumor nodules in the lungs (**Figure 1G**).

### BAL From NSCLC Patients Increased CD3^+^ and CD8^+^ T Cell Infiltration in Lungs

**Figure 2:**
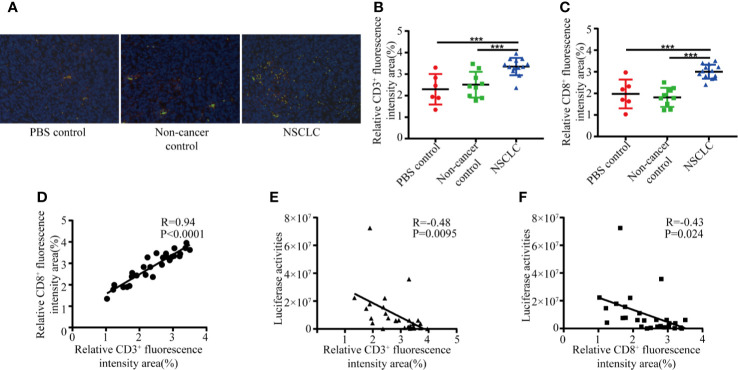
BAL from NSCLC patients increase CD3^+^ and CD8^+^ T cell infiltration. Formalin fixed, paraffin embedded mouse lung sections were stained with CD8 and CD3 antibodies and nuclei were stained with DAPI. **(A)** CD3 (green) and CD8 (red) positive T cell subpopulations. The percentage of positive CD3 **(B)** and CD8 **(C)** were graphed for NSCLC group, NC group and non-cancer control group. Student’s t test, ***p < 0.001; **(D)** Positive correlation between number of CD3 and CD8. The area of positive CD3 **(E)** and CD8 **(F)** was negatively associated with tumor burden.

To examine the mechanisms of how inhalation of BAL inhibits the tumor growth, we performed an immunofluorescence assay to measure the expression of CD3 and CD8 in lung cancer biopsies from tumor-bearing mice. It revealed infiltration of CD3^+^ and CD8^+^ T cells in lung cancer biopsies. Compared to non-cancer controls and PBS groups, NSCLC group showed significantly increased infiltration of CD3^+^ and CD8^+^ T cells (**Figures 2A–C**). We observed a positive correlation between CD3 and CD8 in lung cancer biopsies (**Figure 2D**). The number of infiltrating CD3^+^ and CD8^+^ lymphocytes was negatively associated with the tumor burden (**Figures 2E, F**). It indicated that BAL from NSCLC patients caused inhibition of tumor growth is partly due to immune response.

### BAL Inhalation Altered the Lung Microbiota in Tumor-Bearing Mice

**Figure 3:**
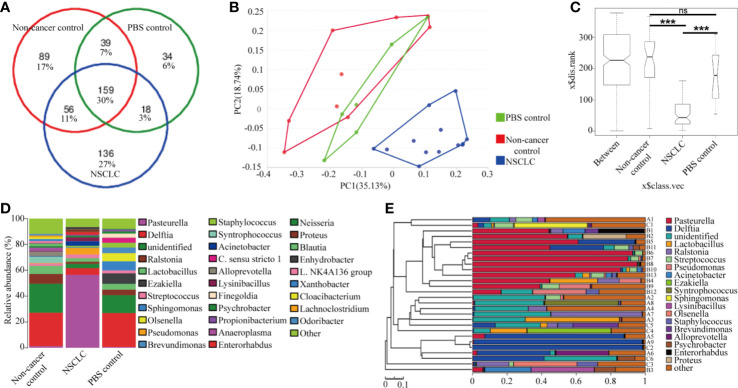
Lung microbiome composition after BAL inhalation. **(A)** Venn diagram representation of the OUT from NSCLC BAL inhalation group, non-cancer control group, and PBS group. The percentage of core lung microbiota genus are shown. **(B)** Principal Component Analysis of OTUs. **(C)** Analysis of similarities (ANOSIM) demonstrated the difference in the lung microbiota between NSCLC group and non-cancer control or PBS group. ***p < 0.001; ns, no significance. **(D)** The composition of major taxonomic groups at genus level in three groups. **(E)** The composition of each sample based on the taxonomic assignment of 16s rDNA sequences. The X-axis represents the abundance of each taxon. A: non-cancer controls; B: NSCLC patients; C: PBS.

Microbes are highly immunogenic that can trigger an immune response. To identify the lung microbiota composition responding to the BAL inhalation, we did 16s rRNA sequencing to examine the differential bacteria colonized in the lungs. The species accumulation curve was used to estimate the number of species in the lungs of mice. The OTUs reached a plateau with the increase of the samples, indicating that the sample size in the study was large enough to reflect richness (Data not shown). A total of 159 genera were detected as core lung microbiota. NSCLC group core lung microbiomes shared 41% of the total OTUs with non-cancer control group and 33% with PBS group (**Figure 3A**). Principal Component Analysis of OTUs (**Figure 3B**) and analysis of similarities (ANOSIM) showed the difference in the lung microbiota between NSCLC group and non-cancer control or PBS group (R = 0.574, p = 0.001; **Figure 3C**). There is no difference in the lung microbiota between non-cancer control and PBS group (R = −0.106, p = 0.842, **Figure 3C**). The microbial communities of NSCLC group were dominated by *Pasteurella*, whereas the microbial communities of non-cancer control and PBS group were dominated by *Delftia* (**Figure 3D**). Based on similarity in genus abundance, cluster analysis was shown in **Figure 3E**. It revealed that NSCLC group differed from the other two groups. Non-cancer controls and PBS group had a high similarity in genus abundance.

### Higher Abundance of *Pasteurella* Was Correlated With Less Tumor Burden

**Figure 4:**
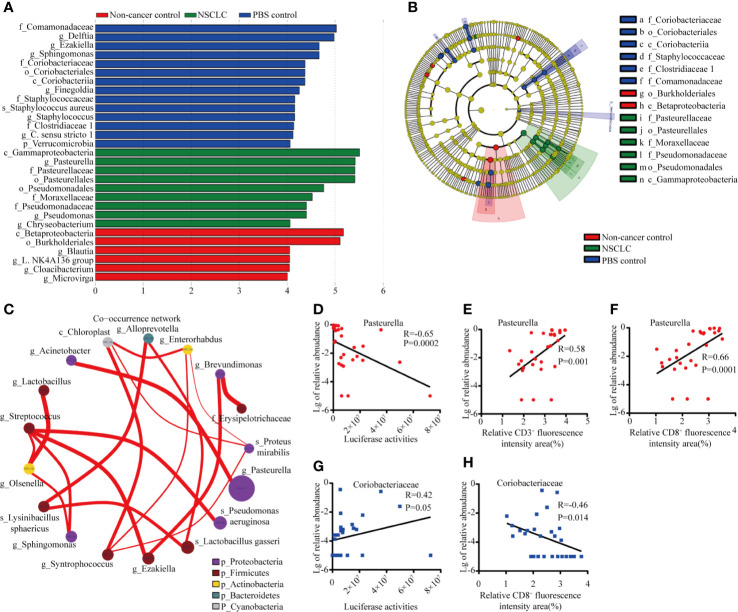
Distinct taxa identified in three groups using LEfSe analysis. **(A)** LDA scores of difference between groups. **(B)** Cladogram shows the enrichment of bacteria in three groups. **(C)** Network of co-occurring microbes was constructed in Cytoscape by correlation data R. Nodes represent microbes, and edges represent the strength of positive correlations between microbes. Correlation between abundance of *Pasteurella* and tumor burden ^#^***p < 0.001; ns, no significance. **(D)**, CD3^+^ T cells **(E)**, and CD8^+^ T cells **(F)**. Correlation between abundance of *Coriobacteriaecae* and tumor burden **(G)** and CD8^+^ T cells **(H)**.

We further identified the differentially abundant genera by LEfSe (Linear discriminant analysis Effect Size) analysis in these three mice groups. As shown in **Figure 4A**, plot from LEfSe analysis displayed LDA score of microbial taxa with significance difference between NSCLC group, non-cancer control group, and PBS group. LDA indicated that the genera *Pasteurella*, *Pseudomonas*, and *Chryseobacterium* were increased in NSCLC group, while genera *Blautia*, *Cloacibacterium*, and *Microvirga* et al. were increased in PBS group (**Figure 4A**). The cladogram showed difference between groups (**Figure 4B**). In the microbiome network constructed from three groups, there were significant positive correlations among the abundance of predominant genera. As shown in **Figure 4C**, *Pasteurella* had positive correlations with *Acinetobacter*. We then analyzed the relationship between *Pasteurella*, *Coriobacteriaceae*, and tumor burden. We found a significant negative correlation between abundance of *Pasteurella* and tumor burden (**Figure 4D**). The abundance of *Pasteurella* was positively correlated with infiltration of CD3^+^ and CD8^+^ T cells, suggesting that *Pasteurella* may trigger the cytotoxic immune response (**Figures 4E, F**). On the contrary, *Coriobacteriaceae* is positively correlated with tumor burden and its abundance was negatively with the number of CD8^+^ T cells (**Figures 4G, H**).

### Microbes Related Cytotoxically Active CD8^+^ TIL Inhibited Tumor Growth

**Figure 5:**
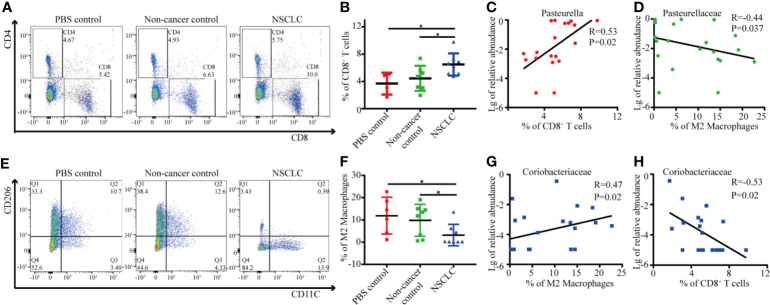
Correlation between immune cells and specific genus in lungs. **(A)** The percentage of CD8+ T cells was determined by flow cytometry. Each dot represents the result for an individual mouse **(B)** Student's t test, *p < 0.05. The correlation between abundance of *Pasteurella* and CD8+ T cells **(C)** and M2 macrophage **(D)** was shown. **(E)** The percentage of M2 macrophages was determined by flow cytometry. Each dot represents the result for an individual mouse **(F)**. Student's t test, *p < 0.05. The correlation between abundance of *Coriobacteriaceae* and CD8+ T cells **(G)** and M2 macrophage **(H)** was shown.

As shown above, BAL inhalation significantly changed the lung microbiota composition and *Pasteurella* was the dominant genus in lung microbiota after inhalation of BAL from NSCLC patients. We then analyzed the relationship between *Pasteurella* and immunologic response. We found a significant increased CD8^+^ T cells in NSCLC group compared to PBS and non-cancer control groups (**Figures 5A, B**). The abundance of *Pasteurella* was positively related with cytotoxic CD8+ TIL and negatively correlated with immunosuppressive M2 (**Figures 5C, D**). We also found a significant decreased M2 macrophages in NSCLC group compared to PBS and non-cancer control groups (**Figures 5E, F**). On the contrary, *Coriobacteriaceae* was negatively with cytotoxic CD8+ TIL and positively with M2 (**Figures 5G, H**).

## Discussion

In this study, we found that mice treated with BAL from lung cancer patients showed an altered microbiota compared to controls. The higher abundance of *Pasteurella* in lungs was correlated with the lower tumor burden. The abundance of *Pasteurella* was positively correlated with infiltration of cytotoxic CD8+ cells and negatively correlated with immunosuppressive M2 macrophages.

Lungs are inhabited by various microbes. Microbial community plays an important role in maintaining the physiological balance of the host, such as helping the host absorb nutrients, resist pathogens, degrade poisons, and regulate the immune system (16, 17).The predominant phyla in healthy lungs are Bacteroidetes, particularly *Prevotella* spp. and *Firmicutes* (18). The most common genera observed in human healthy lungs include *Prevotella*, *Veillonella*, and *Streptococcus* (19). *Staphylococcus* spp., *Bacillus* spp., *Hemophilus influenza*, and *Candida albicans* have been identified in lung cancer patients with inflammation (20). However, mice, regardless whether they were of Specific Pathogen Free (SPF) or non-SPF origin, featured the same high ranking genera, i.e. *Ralstonia*, *Lactobacillus*, *Enterobacteriaceae*, and *Sphingomonas* (21). *Pasteurellaceae*, *Enterobacteria*, and *Firmicutes* were isolated from lungs of non-SPF mice (21). The development of lung cancer is associated with local ecological disturbance and inflammation (22). The dominant genus in PBS treated tumor-bearing mice were *Delftia*, *Ezakiella*, and *Sphingomonas.* The inhalation of BAL from non-cancer controls did not change the community composition of lung microbiota of tumor-bearing mice. However, the inhalation of BAL from NSCLC patients caused a significant shift towards a dominance of *Pasteurella*. We have performed metagenomic sequencing in BAL from 63 individuals and *Pasteurella* spp. has been found with the relative abundance of only 0.003% (OEP000655). The composition of lung microbiota in mice is different from that of the inhaled BAL, suggesting that the growth and colonization of bacteria in lungs may be affected by multiple factors such as immune response, microbial interactions.

Local microbiota trigger inflammation with lung adenocarcinoma by activating lung-resident γδ T cells (22). The absence of symbiotic flora inhibits the development of lung adenocarcinoma (22). In this study, we found that a higher *Pasteurella* in NSCLC BAL inhalation group and the non-cancer control and PBS inhalation group was dominated by *Delftia*. It suggested that inhalation of BAL from lung cancer patients facilitates the growth of *Pasteurella*.

Bacteria and viruses are highly immunogenic that can be used to trigger an immune response towards tumors (23). Bacterial vaccines contain killed or attenuated bacteria that activate the immune system, for example, Tuberculosis vaccine. Several bacterial genera, including *Salmonella*, *Streptococcus*, *Listeria*, *Escherichia* et al., accumulate inside tumors and develop an oncolytic effect (23). Listeria-based tumor vaccines that generates CTL-mediated tumor killing have been presented as an immunotherapy approach in clinical setting (24). The idea of using bacteria as a therapeutic strategy was pioneered by Dr. William Coley, who developed “Coley’s Toxins” to treat cancer patients (25). Mixed Bacterial Vaccines (MBV) that contain *Streptococcus pyogenes* and *Serratia marcescens *kill the tumor cells through induction of immune response. The influence of the gut microbiome on the response to cancer therapy, especially immune checkpoint blockade has received significant attention (26). *Pasteurella* is a facultative anaerobic gram-negative bacterium (GNB). It is a symbiotic microbe that lives in the oropharynx of healthy domestic animals, especially dogs, mice, and cats (27). *Pasteurella* spp. infections may be pathogenic in wild and domestic animals (28). It can spread from animals to humans (29). In humans, *Pasteurella* infection in humans are associated with animal bites or scratches (28). *Pasteurella* vaccines have been used for the prevention of disease in animals. The strong positive relationship between *Pasteurella* and infiltration of CD8+ T cells in lung cancer biopsies shown in this study suggests the promising of using *Pasteurella* as cancer therapeutic vaccine.

The immunosuppressive microenvironment in cancer is also widely reported. Tumor-associated macrophages (TAMs) are the main components of the tumor matrix in a variety of solid tumors (30, 31). In response to microenvironmental stimuli, TAMs polarize towards classically (M1) or alternatively (M2) activated cells, with tumor-inhibitory or tumor-promoting properties, respectively (32). In our findings, M2 macrophages displayed negative association with *Pasteurella* and tumor burden. However, it is not clear the polarization towards to M2 is due to tumor or microbiota dysbiosis.

In conclusion, our findings emphasize the effect of microbial composition on the cancer progression. We demonstrated a significant positive correlation between *Pasteurella* and cytotoxic CD8+ TIL and a negative correlation with M2 macrophages. The abundance of *Pasteurella* was negatively correlated with tumor cell growth. Our findings provide a promising strategy that can be used as a therapeutic vaccine for lung cancer patients.

## Data Availability Statement

The datasets presented in this study can be found in online repositories. The names of the repository/repositories and accession number(s) can be found below: SRA, PRJNA672792.

## Ethics Statement

The studies involving human participants were reviewed and approved by the second hospital of Xiangya, Central South University. The patients/participants provided their written informed consent to participate in this study. The animal study was reviewed and approved by Central South University.

## Author Contributions

The work presented here was carried out as a collaboration between all authors. LZ, JX, NY, and YZ carried out most experiments. JJX, JT, FY, WZ, and MW made contributions to design, analyze data, and interpret data. JJX and LZ have been involved in drafting the manuscript. JJX, JT, WZ, and FY gave most financial support. BS, LW, and LZ collected clinical samples and information. LZ performed statistical analysis. All authors contributed to the article and approved the submitted version.

## Funding

This work was supported by National Natural Science Foundation, China (grant number 81972198, 81773147,81472695); Strategic Priority Research Program of Central South University (ZLXD2017004); Key Research and Development Program of Hunan (2019SK2253).

## Conflict of Interest

The authors declare that the research was conducted in the absence of any commercial or financial relationships that could be construed as a potential conflict of interest.
